# Supplementary material for: Selective Decontamination of the Digestive Tract to Prevent Postoperative Pneumonia and Anastomotic Leakage after Esophagectomy: A Retrospective Cohort Study
Source: Antibiotics (Basel). 2021 Jan 5;10(1):43. doi: 10.3390/antibiotics10010043 (PMC7824731; doi:10.3390/antibiotics10010043)
Supplement: Supplementary file 1 [file antibiotics-10-00043-s001.pdf]

## Supplementary Material

**Table S1. Univariate and multivariate analysis of factors associated with postoperative pneumonia after Ivor Lewis totally minimally invasive esophagectomy.**

| | **Univariate** | | **Multivariate[a]** | |
|---|---|---|---|---|
| | OR (95% CI) | *P* value | OR (95% CI) | *P* value |
| **Patient characteristics** | | | | |
| Sex | | | | |
| Male | *ref* | | | |
| Female | 0.62 (0.35-1.08) | 0.093 | | |
| Age[b] | 1.21 (0.95-1.52) | 0.115 | | |
| Body mass index (kg/m$^2$) | 1.01 (0.97-1.06) | 0.651 | 1.01 (0.96-1.07) | 0.615 |
| ASA classification | | 0.666 | | |
| 1 | *ref* | | | |
| 2 | 1.11 (0.59-2.08) | 0.748 | | |
| ≥3 | 1.31 (0.66-2.59) | 0.437 | | |
| Charlson Comorbidity Index | | 0.115 | | 0.069 |
| 0 | *ref* | | *ref* | |
| 1 | 1.06 (0.68-1.65) | 0.809 | 1.14 (0.70-1.86) | 0.607 |
| ≥2 | 1.68 (1.02-2.77) | <u>0.042</u> | 1.87 (1.10-3.20) | <u>0.021</u> |
| Tumour histology | | 0.371 | | |
| Adenocarcinoma | *ref* | | | |
| Squamous cell carcinoma | 0.81 (0.44-1.50) | 0.507 | | |
| Other | 0.26 (0.03-2.10) | 0.207 | | |
| Tumour stage | | 0.961 | | |
| I | *ref* | | | |
| II | 1.01 (0.63-1.62) | 0.976 | | |
| III | 0.95 (0.56-1.59) | 0.835 | | |
| Tumour location | | 0.161 | | |
| Mid esophagus | *ref* | | | |
| Distal esophagus | 1.00 (0.43-2.35) | 1.000 | | |
| Junction | 1.54 (0.62-3.82) | 0.347 | | |
| **Surgical characteristics** | | | | |
| Year of surgery | | 0.355 | | 0.818 |
| 2012-2013 | *ref* | | *ref* | |
| 2014-2015 | 0.61 (0.34-1.09) | 0.095 | 1.07 (0.54-2.15) | 0.842 |
| 2016-2017 | 0.83 (0.48-1.44) | 0.511 | 1.17 (0.61-2.25) | 0.628 |
| 2018 | 0.86 (0.43-1.72) | 0.667 | 1.500 (0.63-3.58) | 0.361 |
| Duration of surgery[c] | 1.00 (0.94-1.09) | 0.599 | 0.97 (0.89-1.09) | 0.720 |
| Nr. of resected lymph nodes | 1.02 (1.00-1.05) | 0.065 | 1.02 (0.99-1.04) | 0.213 |
| Blood loss (ml)[d] | 1.00 (0.91-1.11) | 0.821 | 1.00 (0.91-1.11) | 0.965 |
| Conversion of surgery | | | | |
| No | *ref* | | | |
| Yes | 0.38 (0.11-1.33) | 0.131 | | |
| Use of SDD | | | | |
| No | *ref* | | *ref* | |
| Yes | 0.43 (0.28-0.66) | <u><0.001</u> | 0.40 (0.23-0.67) | <u><0.001</u> |

*Nr.* number, *SDD* selective decontamination of the digestive tract, *OR* odds ratio, *CI* confidence interval, *ref* reference

[a] Model consists of BMI, Charlson Comorbidity Index, year of surgery, duration of surgery, number of lymph nodes resected, blood loss and use of SDD

[b] OR per 10 years increase in age (per 1 year increase in age: univariate OR 1.019; 95% CI: 0.995-1.043)

[c] OR per 30 minute increase in duration of surgery (per 1 minute increase in surgery: univariate OR 1.000; 95% CI: 0.998-1.003, multivariate OR 0.999; 95% CI: 0.996-1.003)

[d] OR per 100 millilitre increase in blood loss (per 1 millilitre increase in blood loss: univariate OR 1.000; 95% CI: 0.999-1.001, multivariate OR 1.000; 95% CI: 0.999-1.001)

**Table S2. Univariate and multivariate analysis of factors associated with anastomotic leakage after Ivor Lewis totally minimally invasive esophagectomy.**

| | **Univariate** | | **Multivariate[a]** | |
|---|---|---|---|---|
| | OR (95% CI) | *P* value | OR (95% CI) | *P* value |
| **Patient characteristics** | | | | |
| Sex | | | | |
| Male | *ref* | | | |
| Female | 0.61 (0.29-1.27) | 0.184 | | |
| Age[b] | 1.07 (0.80-1.42) | 0.655 | | |
| Body mass index (kg/m$^2$) | 1.05 (0.99-1.11) | 0.116 | 1.04 (0.98-1.10) | 0.188 |
| ASA classification | | 0.581 | | |
| 1 | *ref* | | | |
| 2 | 1.42 (0.61-3.30) | 0.419 | | |
| ≥3 | 1.62 (0.65-4.00) | 0.299 | | |
| Charlson Comorbidity Index | | 0.963 | | |
| 0 | *ref* | | | |
| 1 | 1.00 (0.59-1.72) | 0.990 | | |
| ≥2 | 0.92 (0.48-1.77) | 0.796 | | |
| Tumour histology | | 0.365 | | |
| Adenocarcinoma | *ref* | | | |
| Squamous cell carcinoma | 0.86 (0.39-1.90) | 0.706 | | |
| Other | 2.63 (0.64-10.78) | 0.179 | | |
| Tumour stage | | 0.895 | | |
| I | *ref* | | | |
| II | 1.02 (0.57-1.83) | 0.948 | | |
| III | 0.89 (0.47-1.71) | 0.733 | | |
| Tumour location | | 0.387 | | |
| Mid esophagus | *ref* | | | |
| Distal esophagus | 2.70 (0.62-11.69) | 0.184 | | |
| Junction | 2.89 (0.63-13.18) | 0.171 | | |
| **Surgical characteristics** | | | | |
| Year of surgery | | 0.466 | | 0.521 |
| 2012-2013 | *ref* | | *ref* | |
| 2014-2015 | 0.60 (0.30-1.20) | 0.146 | 0.81 (0.38-1.69) | 0.565 |
| 2016-2017 | 0.79 (0.41-1.52) | 0.484 | 0.75 (0.39-1.46) | 0.396 |
| 2018 | 0.61 (0.25-1.47) | 0.268 | 0.49 (0.19-1.25) | 0.135 |
| Duration of surgery[c] | 1.06 (1.00-1.16) | 0.109 | 1.00 (1.00-1.01) | 0.087 |
| Nr. of resected lymph nodes | 1.00 (0.97-1.03) | 0.889 | | |
| Blood loss (ml)[d] | 1.00 (0.91-1.11) | 0.942 | | |
| Conversion of surgery | | | | |
| No | *ref* | | | |
| Yes | 0.55 (0.13-2.42) | 0.429 | | |
| Use of SDD | | | | |
| No | *ref* | | *ref* | |
| Yes | 0.48 (0.28-0.83) | 0.009 | 0.46 (0.26-0.84) | 0.011 |

*Nr.* number, *SDD* selective decontamination of the digestive tract, *OR* odds ratio, *CI* confidence interval, *ref* reference

[a] Model consists of BMI, year of surgery, duration of surgery and use of SDD

[b] OR per 10 years increase in age (per 1 year increase in age: univariate OR 1.007; 95% CI: 0.987-1.036)

[c] OR per 30 minute increase in duration of surgery (per 1 minute increase in surgery: univariate OR 1.002; 95% CI: 1.000-1.005, multivariate OR 1.003; 95% CI: 1.000-1.006)

[d] OR per 100 millilitre increase in blood loss (per 1 millilitre increase in blood loss: univariate OR 1.000; 95% CI: 0.999-1.001)

**Table S3. Univariate and multivariate analysis of factors associated with all-cause 1-year mortality after Ivor Lewis totally minimally invasive esophagectomy.**

| | **Univariate** | | **Multivariate**[a] | |
|---|---|---|---|---|
| | OR (95% CI) | *P* value | OR (95% CI) | *P* value |
| **Patient characteristics** | | | | |
| Sex | | | | |
| Male | *ref* | | | |
| Female | 0.83 (0.45-1.53) | 0.543 | | |
| Age[b] | 1.38 (1.06-1.81) | 0.016 | 1.36 (1.02-1.81) | 0.037 |
| Body mass index (kg/$m^2$) | 1.020 (0.97-1.07) | 0.457 | | |
| ASA classification | | 0.026 | | |
| 1 | *ref* | | | |
| 2 | 0.87 (0.43-1.75) | 0.692 | | |
| ≥3 | 1.68 (0.80-3.56) | 0.173 | | |
| Charlson Comorbidity Index | | 0.010 | | 0.065 |
| 0 | *ref* | | *ref* | |
| 1 | 1.59 (0.96-2.64) | 0.074 | 1.30 (0.75-2.25) | 0.345 |
| ≥2 | 2.31 (1.32-4.02) | 0.003 | 2.01 (1.12-3.60) | 0.020 |
| Tumour histology | | 0.783 | | |
| Adenocarcinoma | *Ref* | | | |
| Squamous cell carcinoma | 0.77 (0.37-1.61) | 0.492 | | |
| Other | 1.08 (0.22-5.47) | 0.922 | | |
| Tumour stage | | 0.001 | | 0.009 |
| I | *ref* | | *ref* | |
| II | 2.93 (1.53-5.64) | 0.001 | 2.71 (1.39-5.29) | 0.004 |
| III | 3.68 (1.85-7.33) | <0.001 | 2.89 (1.36-6.12) | 0.006 |
| Tumour location | | 0.815 | | |
| Mid esophagus | *ref* | | | |
| Distal esophagus | 1.15 (0.45-2.93) | 0.775 | | |
| Junction | 0.97 (0.35-2.69) | 0.956 | | |
| **Surgical characteristics** | | | | |
| Year of surgery | | 0.049 | | |
| 2012-2013 | *ref* | | | |
| 2014-2015 | 1.36 (0.73-2.55) | 0.338 | | |
| 2016-2017 | 0.74 (0.39-1.41) | 0.361 | | |
| 2018 | 1.91 (0.75-4.87) | 0.173 | | |
| Duration of surgery[c] | 1.09 (1.00-1.16) | 0.026 | 1.06 (1.00-1.16) | 0.073 |
| Nr. of resected lymph nodes | 0.99 (0.97-1.02) | 0.618 | | |
| Blood loss (ml)[d] | 0.90 (0.82-1.00) | 0.262 | | |
| Conversion of surgery | | | | |
| No | *ref* | | | |
| Yes | 2.29 (0.91-5.76) | 0.078 | | |
| Use of SDD | | | | |
| No | *ref* | | *ref* | |
| Yes | 1.53 (0.99-2.38) | 0.057 | 1.31 (0.80-2.14) | 0.278 |

*Nr.* number, *SDD* selective decontamination of the digestive tract, *OR* odds ratio, *CI* confidence interval, *ref* reference

[a] Model consists of age, Charlson Comorbidity Index, tumour stage, duration of surgery and use of SDD.

[b] OR per 10 years increase in age (per 1 year increase in age: univariate OR 1.033; 95% CI: 1.006-1.061, multivariate OR 1.312; 95% CI: 1.002-1.061)

[c] OR per 30 minute increase in duration of surgery (per 1 minute increase in surgery: univariate OR 1.003; 95% CI: 1.000-1.005, multivariate OR 1.002; 95% CI: 1.00-1.005)

[d] OR per 100 millilitre increase in blood loss (per 1 millilitre increase in blood loss: univariate OR 0.999; 95% CI: 0.998-1.000)
